# Histopathological Features and Differential Diagnosis of Pityriasis Lichenoides Et Varioliformis Acuta: A Retrospective Study of 12 Cases

**DOI:** 10.7759/cureus.109743

**Published:** 2026-05-27

**Authors:** Zhen Yue, Congjun Jiang, Wanlu Zhang, Gege Zhu, Huiya Sun

**Affiliations:** 1 Department of Dermatology, The First Affiliated Hospital of Bengbu Medical University, Bengbu, CHN

**Keywords:** differential diagnosis, histopathology, pityriasis lichenoides et varioliformis acuta, pleva, retrospective study

## Abstract

Objective: This retrospective study aimed to describe the clinicopathological features of pityriasis lichenoides et varioliformis acuta (PLEVA) in a cohort of 12 patients, to evaluate the correlation between clinical morphology and key histopathological findings, and to assess the diagnostic utility of these features for improving differential diagnosis from its main mimics.

Methods: Twelve patients with biopsy-proven PLEVA were seen in our dermatology department between June 2023 and October 2025. Slides were reviewed by two senior dermatopathologists independently, without prior knowledge of the presumptive diagnosis; final diagnoses were confirmed through clinicopathological correlation. Key histopathological features and differential diagnoses were reviewed against the published literature.

Results: The patients included nine males and three females (male-to-female ratio 3:1), with a mean age of 23.5 ± 8.5 years (range 11-37 years). Lesions presented as scattered erythematous to brown papules with adherent scales or crusts; central necrosis was observed in five cases (42%). Histopathological analysis revealed hyperkeratosis, basal cell liquefaction degeneration, and superficial perivascular/interface lymphocytic infiltration in 100% of cases. Parakeratosis was noted in 92%, spongiosis in 67%, extravasated erythrocytes in 75%, and pigment-laden macrophages in 42%. No leukocytoclastic vasculitis or Pautrier microabscesses were identified. Clinical and histological findings correlated well in most cases; in one atypical case presenting only with brown macules, the clinical history was essential for reaching the correct diagnosis.

Conclusion: PLEVA is marked by prominent interface dermatitis and extravasation of erythrocytes. A thorough clinicopathological correlation is essential to differentiate it from various other inflammatory and lymphoproliferative dermatological conditions.

## Introduction

Pityriasis lichenoides et varioliformis acuta (PLEVA), also referred to as Mucha-Habermann disease, is the acute subtype present in the spectrum of pityriasis lichenoides [1,2]. Patients typically develop recurrent crops of erythematous to reddish-brown papules that often go on to centrally necrose and crust, or varioliform scar, particularly on the trunk and flexures of the limbs.

Although the precise trigger remains unclear, infection and other immune stimuli have been implicated in initiating the response. The resulting CD8+ T-cell-mediated injury to keratinocytes leads to their apoptosis and the characteristic interface dermatitis [1,3]. Histologically, PLEVA is characterized by interface dermatitis accompanied by superficial dermal lymphocytic infiltration and frequent erythrocyte extravasation [3,4]. An accurate clinical diagnosis of this lesion requires the clinical setting and histopathology due to its resemblance to guttate psoriasis, pityriasis rosea, lichen planus, lichenoid drug eruption, secondary syphilis, lymphomatoid papulosis, and early mycosis fungoides (MF) [4,5]. As stated in published case series, clinical and pathological features of PLEVA are known [6,7].

We retrospectively reviewed 12 biopsy-proven cases of PLEVA in our hospital to describe the clinicopathological features of the disease, evaluate the correlation between clinical morphology and key histopathological findings, and assess the utility of these features in differential diagnosis.

## Materials and methods

Study design

This was a retrospective and descriptive study. We reviewed the clinical records and biopsy slides of 12 patients diagnosed with PLEVA in our dermatology department from June 2023 to October 2025.

Patient selection

Inclusion criteria were recurrent papular lesions with scales or crusts, along with histopathology results consistent with PLEVA and complete clinical and pathological information. Patients with ambiguous diagnoses or concurrent skin disorders that might influence interpretation were not included.

Histopathological evaluation

Biopsies were obtained from representative active lesions on the trunk and extremities, with a preference for fully developed papules or plaques showing adherent scales or central necrosis. When lesions at different evolutionary stages were present, the most clinically active lesion was preferentially biopsied. Skin biopsy specimens were fixed in 10% neutral buffered formalin, embedded in paraffin, and sectioned at 4 μm for hematoxylin-eosin staining. Slides were reviewed by two senior dermatopathologists independently; neither was informed of the presumptive clinical diagnosis beforehand, keeping the histological assessment unbiased. Final diagnoses were then established through clinicopathological correlation. Where the two evaluators disagreed, the cases were re-examined jointly until a consensus was reached. The Olympus CX31 microscope (Olympus Corporation, Tokyo, Japan) was used to record all epidermal and dermal changes. The histopathological features analyzed were selected based on those most consistently described and considered diagnostically relevant in established studies on PLEVA [3,8].

Statistical analysis

Statistical analysis was performed using IBM SPSS Statistics for Windows, Version 26 (Released 2018; IBM Corp., Armonk, New York, United States). Continuous variables are presented as mean ± standard deviation (SD) or median (range), and categorical variables are expressed as frequencies and percentages. Due to the small sample size and the purely descriptive nature of the study, no inferential statistical tests were conducted.

## Results

Clinical features

The patients included nine males and three females (male-to-female ratio 3:1), with a mean age of 23.5 ± 8.5 years (range 11-37 years) (Table 1). The mean disease duration was 5.4 ± 7.2 months (range 0.5-24 months). Lesions involved the trunk and extremities with a predilection for flexural areas; palms, soles, and mucous membranes were spared - consistent with the known distribution of PLEVA [1,2]. Lesions typically measured 2-10 mm and presented as erythematous to brown papules with adherent scales or crusts. Central necrosis was observed in five cases (42%), and varioliform scarring in three resolving lesions (25%). Auspitz sign was negative in all cases. Half the patients reported mild pruritus; the remainder had none. Biopsy sites reflected the lesion distribution: trunk in seven cases (58%; lumbar region five, upper back one, abdomen one), upper extremity flexural surface in three (25%), and lower extremity in two (17%) (Table 2).

**Table 1:** Demographic characteristics of the 12 patients with PLEVA PLEVA: pityriasis lichenoides et varioliformis acuta

Characteristic	Value
Number of patients	12
Male	9 (75.0%)
Female	3 (25.0%)
Mean age (years)	23.5 ± 8.5 (range 11-37)
Mean disease duration (months)	5.4 ± 7.2 (range 0.5-24)

**Table 2:** Clinical features and biopsy site distribution in 12 patients with PLEVA This table summarizes the distribution of lesion morphology and associated symptoms observed in the 12 patients. PLEVA: pityriasis lichenoides et varioliformis acuta

Feature	Number of cases	Percentage (%)	Notes
Biopsy site			
Trunk	7	58	Lumbar region (five), back (one), abdomen (one)
Upper extremity	3	25	Flexural surface
Lower extremity	2	17	Flexural surface (one), inner thigh (one)
Mucosal involvement	0	0	None
Erythematous to brown papules	12	100	All cases
Adherent scales or crusts	12	100	Characteristic feature
Central necrosis	5	42	Present in some cases
Varioliform scarring	3	25	Seen in resolving lesions
Auspitz sign	0	0	Negative in all cases
Pruritus (mild)	6	50	Absent in 50% of patients

Histopathological features

The biopsies demonstrated broadly similar inflammatory patterns with variable expression of individual histopathological features. These changes are consistent with those reported in earlier case series and reviews [3,8].

The epidermal alterations recorded hyperkeratosis in all cases (100%), mostly next to parakeratotic areas (92%). In seven cases, parakeratosis was focal and irregular with underlying granular layer thinning, forming the typical “skip” pattern [3,8]. One atypical case showed minimal parakeratosis and predominantly scattered brown macules, findings most consistent with resolving post-inflammatory hyperpigmentation [9]. Mild to moderate spongiosis involving the lower epidermis was observed in 67% of cases. All the biopsies (100%) showed basal cell liquefaction degeneration, with some having keratinocyte necrosis and adnexal involvement [3,8]. The absence of Munro microabscesses and true Pautrier microabscesses was a key feature used to differentiate PLEVA from psoriasis and MF [4,5].

Dermal Changes

Dense superficial perivascular infiltration of lymphocytes was observed in 100% of cases. The infiltrate commonly extended to the dermoepidermal junction. A total of 75% of cases exhibited extravasated erythrocytes, and 42% of cases had pigment-laden macrophages. There were no signs of leukocytoclastic vasculitis [3]. Both epidermal and dermal changes are summarized in Table 3.

**Table 3:** Histopathological characteristics in 12 patients with PLEVA (n=12) This table summarizes the frequency and main characteristics of key histopathological features observed in the 12 patients. PLEVA: pityriasis lichenoides et varioliformis acuta

Pathological feature	Number of cases	Percentage (%)	Main characteristics
Hyperkeratosis	12	100	Focal, adjacent to parakeratotic areas
Parakeratosis	11	92	Irregular focal distribution, alternating with hyperkeratosis
Spongiosis	8	67	Mild to moderate, mainly lower epidermis
Lymphocytic infiltration (superficial dermis)	12	100	Perivascular cuff-like, extending to the dermoepidermal junction
Liquefaction degeneration of basal cells	12	100	Mild to moderate interface dermatitis
Extravasated erythrocytes	9	75	Papillary dermis, indicating vascular damage
Pigment-laden macrophages	5	42	Superficial dermis, reflecting prior hemorrhage
Leukocytoclastic vasculitis	0	0	None observed

Clinicopathological correlation

Clinical and histopathological findings were concordant in most cases. The five patients with central necrosis or hemorrhagic crusting each showed prominent keratinocyte necrosis and epidermal disruption on biopsy. Adherent mica-like scales corresponded to hyperkeratosis with focal skip parakeratosis on histology. Among the nine cases with erythrocyte extravasation on histology, hemorrhagic crusting or purpuric discoloration was visible clinically at lesion centers in each. The one case with brown macules showed reduced parakeratosis and predominant pigment-laden macrophages, with no active epidermal changes - findings discussed in the context of the atypical case in the Discussion section.

**Figure 1:**
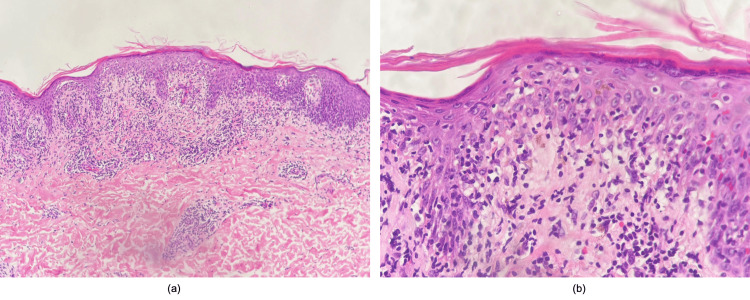
Representative histopathological images of PLEVA (a) Focal hyperkeratosis with parakeratosis, epidermal spongiosis, and superficial perivascular lymphocytic infiltration (HE, ×100). (b) Basal cell liquefaction degeneration, papillary dermal lymphocytic infiltrate, and extravasated erythrocytes (HE, ×400). PLEVA: pityriasis lichenoides et varioliformis acuta

Representative histopathological images demonstrating these key features are shown in Figure 1. Figure 1a shows focal hyperkeratosis with parakeratosis, epidermal spongiosis, and superficial perivascular lymphocytic infiltration at ×100 magnification. Figure 1b demonstrates basal cell liquefaction degeneration, papillary dermal lymphocytic infiltrate, and extravasated erythrocytes at ×400 magnification.

## Discussion

Our series demonstrated a fairly consistent pattern of interface dermatitis associated with erythrocyte extravasation. Though characteristic, these features are not pathognomonic, hence dependent on clinicopathological correlation [1,2].

Pathophysiological basis and key features

The histopathological findings are consistent with cytotoxic T-cell-mediated injury at the dermoepidermal junction, resulting in keratinocyte apoptosis, basal liquefactive degeneration, and interface dermatitis [1,3]. In our series, erythrocyte extravasation was identified in 75% of cases, often accompanied by pigment-laden macrophages, findings that likely reflect vascular injury and prior hemorrhage [3,8]. The characteristic “skipping” pattern of parakeratosis and hyperkeratosis may reflect disordered epidermal maturation and was frequently associated with basal liquefactive degeneration and superficial perivascular lymphocytic inflammation, consistent with the interface inflammatory pattern of PLEVA [3,8].

One case in our series was clinically and histopathologically atypical, presenting predominantly with macular brown lesions and minimal parakeratosis. Although this appearance is consistent with post-inflammatory hyperpigmentation [9], the diagnosis of PLEVA was established based on the patient’s history of recurrent crops over several months, the presence of other typical lesions, and supportive histopathological findings including interface dermatitis and pigment-laden macrophages. This case most likely represents the resolving phase of PLEVA, as reported in previous clinicopathological series [2,7]. This case was included in the overall cohort but also evaluated separately to avoid skewing the frequency data of acute-phase features: parakeratosis was absent, pigment-laden macrophages predominated in the papillary dermis, and the interface pattern was attenuated but still identifiable - findings consistent with the post-inflammatory stage. Its inclusion reflects the polymorphic nature of PLEVA, where lesions at different stages frequently coexist in the same patient [1,2].

These histopathological features correlated well with the corresponding clinical findings and provided useful diagnostic clues. Interface dermatitis typically manifests clinically as erythematous papules with central necrosis or crusting. This clinical appearance may suggest the diagnosis of PLEVA before histopathological confirmation. Erythrocyte extravasation is particularly useful in narrowing the differential: its clinical expression as hemorrhagic crusting or purpuric discoloration at lesion centers is not seen in pityriasis rosea or guttate psoriasis [6,10], providing a useful clue for clinical differentiation. The resolving phase presents a different picture: pigment-laden macrophages dominate, epidermal activity is reduced, and the interface pattern becomes attenuated [7]. Our atypical case also highlights the importance of biopsy timing, as resolving lesions may lack the characteristic acute histopathological features of PLEVA [7,8], and without the clinical context, such a biopsy can easily be misread.

Differential diagnosis

PLEVA shares features with several inflammatory and lymphoproliferative disorders and must be carefully distinguished [4,5]. Guttate psoriasis is marked by frequent acanthosis, granular layer thinning, Munro microabscesses, as well as broadened papillary capillaries, without a noticeable interface alteration. The parakeratosis in guttate psoriasis is usually quite diffuse and regular. This is unlike the focal “skip” pattern seen in our cases [9,10].

Pityriasis rosea may show focal parakeratosis and spongiosis but usually lacks significant basal liquefaction degeneration and erythrocyte extravasation. Typically, it goes away all on its own within six to eight weeks [6,10].

Lichen planus displays a band-like infiltrate of lymphocytes, with marked basal liquefaction plus saw-tooth hyperplasia and wedge-shaped hypergranulosis. In contrast, our cases displayed pronounced focal parakeratosis with minimal, non-band-like infiltration [3].

Early MF may closely mimic PLEVA because both can demonstrate epidermotropism. However, Pautrier microabscesses, significant lymphocytic atypia, and denser infiltrates favor MF. Our cases did not show significant atypia or true Pautrier collections. There may be transient clonality in PLEVA, while persistent clonality supports MF. The prevalence of CD8+ in PLEVA compared to that of CD4+ in MF is also useful [4,5,11]. Atypical lesions typically require repeated biopsy and long-term follow-up [7].

A lichenoid drug eruption is PLEVA’s histological twin. They differ in that lichenoid drug eruption has more eosinophils, prominent keratinocyte necrosis, deeper perivascular involvement, and more extensive interface changes. Careful review of the medication history is important for differentiation [12,13].

The characteristics of secondary syphilis include psoriasiform hyperplasia, perivascular infiltrates consisting of plasma cells, and the swelling of the endothelium. Diagnosis can be confirmed by special stains or serology [14]. Lymphomatoid papulosis contains numerous atypical lymphocytes, a varied background of inflammatory cells that exhibit abundant apoptosis with numerous CD30+ cells [15,16].

To provide a more intuitive comparison of the pathological features between PLEVA and its major differential diseases, Table 4 summarizes the key distinguishing points.

**Table 4:** Comparison of pathological features between PLEVA and major differential diseases Key differential histopathological features are referenced in the main text [4,5,9,10,12-16]. PLEVA: pityriasis lichenoides et varioliformis acuta

Disease	Key histopathological features	Key points for differential diagnosis from PLEVA
PLEVA	Focal parakeratosis, basal liquefaction degeneration, superficial perivascular lymphocytic infiltrate, extravasated erythrocytes, pigment-laden macrophages	Prominent interface dermatitis, erythrocyte extravasation, no atypia or Pautrier microabscesses
Guttate psoriasis (GP)	Regular acanthosis, Munro microabscesses, dilated papillary capillaries	Neutrophilic aggregates, absent interface changes [9,10]
Pityriasis rosea (PR)	Focal parakeratosis, mild spongiosis ± microvesicles	Minimal basal liquefaction, rare extravasation, self-limited course [6,10]
Lichen planus (LP)	Dense band-like infiltrate, wedge hypergranulosis, Civatte bodies	Absent parakeratosis, prominent Civatte bodies [3]
Mycosis fungoides (MF)	Epidermotropism, Pautrier microabscesses, atypical lymphocytes	Cellular atypia, persistent clonality [4,5]
Lichenoid drug eruption	Similar to PLEVA, often with eosinophils and necrosis	Medication history, increased eosinophils [12,13]
Secondary syphilid	Plasma cell-rich perivascular infiltrate, endothelial swelling	Plasma cells, positive serology [14]
Lymphomatoid papulosis (LyP)	Atypical lymphocytes, numerous CD30+ cells	Marked atypia, CD30 positivity [15,16]

A few methodological choices in this study merit brief comment. Feature frequencies were tabulated systematically across all 12 cases (Table 3), making it straightforward to see which histological findings are reliable and which are variable - a level of detail not easily obtained from narrative case reports. Table 4 offers a side-by-side comparison of PLEVA with its seven main histological mimics, intended as a practical reference at the microscope. Negative findings, including the absence of Munro microabscesses, Pautrier collections, and leukocytoclastic vasculitis, were diagnostically useful in differentiating PLEVA from its mimics.

Therapeutic and prognostic implications

Therapy is selected based on how severe and extensive the illness is. Mild localized PLEVA can often be treated with topical mid-strength steroid or calcineurin inhibitors. Narrowband ultraviolet B phototherapy usually works well for widespread lesions [2]. In resistant instances, erythromycin or tetracycline systemic antibiotics or methotrexate may be required [17]. Dupilumab has shown potential benefit in isolated reports, although supporting evidence remains limited [18].

Usually benign, PLEVA often has a chronic relapsing course. Long-term follow-up is advisable due to rare reports of progression to MF [7]. In children and adolescents, prolonged activity and residual hypopigmentation occur commonly, indicating that they must be observed further [2]. For atypical or persistent lesions, it is reasonable to repeat the biopsy, perform immunohistochemistry, and do T-cell receptor gene rearrangement [7,11].

Study limitations

The small sample size and single-center retrospective design may affect the results of this study. Immunohistochemical and molecular tests were not performed, limiting our ability to analyze immunophenotype and clonality in depth. It is essential to conduct larger, multicenter prospective studies [10,11].

## Conclusions

PLEVA is characterized by interface dermatitis with frequent erythrocyte extravasation and superficial lymphocytic infiltration. Accurate diagnosis depends on careful clinicopathological correlation, particularly in distinguishing PLEVA from early MF, guttate psoriasis, and lichenoid dermatoses. Although generally benign, atypical or persistent lesions warrant long-term follow-up and, when necessary, repeat biopsy evaluation.
